# The global condition of higher education students’ executive function research: a scoping review

**DOI:** 10.3389/fpsyg.2026.1636067

**Published:** 2026-03-11

**Authors:** Lorette Pretorius, Marita M. Heyns

**Affiliations:** Optentia Research Unit, Northwest University, Vanderbijlpark, South Africa

**Keywords:** academic achievement, executive function, higher education students, international context, scoping review

## Abstract

The fundamental cognitive processes known as executive functions (EF) allow people to control their thoughts, feelings, and actions to engage in goal-directed behaviour. Strong EF abilities are linked to better academic achievement and student success in higher education (HE). This scoping review sought to map the volume and nature of scholarly articles published between 2014 and 2024 that address undergraduate students’ EF skills in HE worldwide. The databases of Web of Science, Scopus, and EBSCOHost were thoroughly searched. The review adhered to the scoping review process developed by the Joanna Briggs Institute (JBI). The inclusion criteria were met by 28 articles after 8,237 records were screened. The literature indicates a sharp rise in scholarly attention in the last 3 years, increasingly favouring multidisciplinary work. The association between EF and academic achievement is the focus of many studies, which use quantitative approaches. Most research is carried out in nations in the northern hemisphere and frequently incorporates viewpoints from other disciplines. The conceptual and methodological complexity in this topic is highlighted by the variety of EF components studied and the breadth of assessment techniques employed. To develop successful educational interventions and support measures, our findings emphasise the need for more inclusive, international, and diverse approaches to the research of EF in HE.

## 1 Introduction and background

Higher education (HE) students face many difficulties, with cognitive-demanding settings being one of the main barriers to success. A key issue lies in students’ challenge to apply self-regulation, task organisation, flexibility (Stasolla et al., 2025; Zimmerman, 2002), autonomy, independent decision-making (Knouse et al., 2014) and accountability and adaptability (Dias et al., 2022). To encourage these cognitive skills in HE, institutions need a clear conceptualisation of executive function (EF), the mental processes that regulate thinking and behaviour (Gray-Burrows et al., 2019), which will guide the implementation of support strategies in programmes.

Research on EF continues to increase, with many studies in education, specifically in early childhood and adolescence. Recent studies provide valuable insights into how EFs are represented in educational approaches (Dias et al., 2022; Häsä et al., 2024; Manuhuwa et al., 2024), with a lack of studies in the HE context, globally. Therefore, a thorough synthesis is necessary to create awareness of EF research in HE to determine how EF is measured and to identify the factors impacting students’ EF, aiming to enhance learning, research, and academic performance.

Without a synthesis, we risk the consequence of emotional issues like stress, anxiousness, and depression, affecting EF skills (concentration, planning, inhibitory control, task initiation, and organisation), causing undergraduate students to drop out of their studies (Wijbenga et al., 2024).

EF research is complicated, involving advanced mental functions essential for achieving goals, behaviour change, and academic performance (Gray-Burrows et al., 2019). High levels of cognitive functioning and overall well-being of students is essential for student success in HE settings. These cognitive abilities enable individuals to regulate thoughts, emotions, and actions to achieve goals (Diamond, 2013; Romero-López et al., 2021). EF plays a fundamental role in facilitating self-regulation, time management, and cognitive flexibility in the challenging environment of HE, where students must manage deadlines, participate in autonomous learning, and balance conflicting duties (Diamond, 2013; Roebers, 2017; Zelazo et al., 2016; Zimmerman, 2002). The cognitive basis of self-regulated learning is EF, which gives students the ability to organise, regulate, and modify their learning in structured settings (Zimmerman, 2002). EFs serve as vital skills, which significantly impacts a person’s achievement in different aspects of life. In addition, EFs promote physical and mental wellness, social–emotional growth, and other significant aspects in life (Bunge, 2024). EF development is vital from preschool through to high school, and eventually to tertiary education, shifting from foundational knowledge to higher-order thinking (Corcoran and O’Flaherty, 2017). Since HE is more rigorous and unstructured than high school, having EF abilities are essential for success (Pinochet-Quiroz et al., 2022).

Research indicates that intricate neural systems in the pre-frontal cortex are where EF operations occur, and these networks communicate with other brain regions to produce new connections, resulting in enhanced EF (Spreij et al., 2023). The pre-frontal cortex, a vulnerable part of the human brain, is the most susceptible to poor health, depression, loneliness, and anxiousness, which affects EFs (Diamond and Ling, 2016). The authors further emphasise that the opposite is true; when individuals are healthy, calm, peaceful, and emotionally and socially in a good place, EFs are functioning well (Diamond and Ling, 2016).

EF, also referred to as cognitive control, comprises core competencies such as cognitive flexibility (CF), working memory (WM), and inhibitory control (IC; Diamond, 2013; Roebers, 2017; Zelazo et al., 2016). CF refers to the ability to shift perspectives in response to changing demands and includes components such as cognitive reframing and flexible problem-solving (Dennis and Vander Wal, 2010; Toraman et al., 2020). When an approach to problem-solving fails, CF is needed to think of or to come up with alternative approaches (Diamond and Ling, 2016) to deal with the particular situation. WM enables the temporary storage and manipulation of information (Gray-Burrows et al., 2019; Miyake et al., 2000), and refers to the capacity to execute complex cognitive operations such as reasoning, solving problems, and comprehending language (Amzil, 2022). Moreover, the presence of IC empowers individuals to modify and determine their responses and actions instead of adhering to established habits, and supports behavioural control and focused attention (Diamond, 2013). These interrelated processes are central to navigating the academic challenges of HE (Follmer, 2021; Ramos-Galarza et al., 2019). Strong EF abilities are increasingly linked to academic success, perseverance, and student engagement (Józsa et al., 2022; Ramos-Galarza et al., 2019).

Regardless, the importance of EF for student performance and how these abilities are measured in HE remains a challenge.

EF measurement in HE is still a contentious topic and presents a significant gap in existing research. According to research, assessing EF in HE students generally encompasses diverse methodologies, mainly classified into self-report questionnaires and performance-based tests. Self-report questionnaires allow students to rate their own EF skills or challenges in real-world scenarios (Rabin et al., 2011; Ramos-Galarza et al., 2019). Follmer (2021) argues that self-report assessments are known to have limitations, including the possibility of bias. Although performance-based assessments provide objective information, they might not accurately represent daily functioning (Manuhuwa et al., 2024). Research seldom includes performance-based and self-report methodologies at the same time to balance their strengths and gaps. Additionally, the neuropsychological processing concerns checklist is used by educators to rate WM and EF items, among other elements of student competence linked to academic performance (Manuhuwa et al., 2024). Although it offers data on how well students are performing, this is a teacher report and not a direct means of student assessment. It is noted that both measures reflect little integration, indicating that both approaches collect behaviour related data.

Studies on EF, specifically focusing on WM and CF have dramatically affected our knowledge about how individuals grow, cognition, and psychological wellness (Amzil, 2022). Extensive research by Diamond and Ling (2016) report on 84 intervention studies regarding effective methods to improve EFs. These revelations highlight how crucial it is to comprehend not only whether EF matters in HE but also how it may be adequately measured and encouraged. Despite their significance, little comprehensive research has focused specifically on EF in undergraduate populations. Specifically, no additional scoping reviews have been performed on this topic. Therefore, this study focuses on undergraduate students, often in their early adult years, a crucial time for EF development since EF improvement often target children and teenagers, with a clear lack of awareness among HE students.

This gap largely influences EF comprehension in HE. The need for a comprehensive synthesis of the literature is illustrated by these methodological discrepancies, the predominance of EF research on children, the narrow geographic diversity of studies, and a lack of longitudinal designs. This emphasises the urgency for studies that apply these conclusions to HE contexts (Dias et al., 2022). HE places a substantial amount of pressure on EFs and strong EFs will be essential in helping students adjust to these expectations (Knouse et al., 2014). Concentrating on EFs give them the cognitive skills they need for life beyond their studies. Therefore, this review seeks to provide a comprehensive overview of how EF skills have been studied among undergraduate students in HE. We aim to answer the following question: “What has been revealed by research on EF in terms of knowledge gaps, study designs, types of measurements, and contextual factors in the HE context globally?”

## 2 Materials and methods

The Right Review tool (Amog et al., 2022) was used to confirm that a scoping review is a proper fit for the current study’s aim. Scoping reviews provide a comprehensive overview of the evidence on a topic, regardless of research quality, and are valuable for analysing developing topics, clarifying essential concepts, and identifying gaps. A scoping review is worthy when a researcher aims to find research gaps, determine and inform potential future research fields, encompass a body of knowledge and define or summarise ideas and themes, as it is with the current research study (Munn et al., 2018; Peters et al., 2015, 2021). Moreover, scoping reviews are most effective when the research question entails investigating, discovering, mapping, reporting or addressing traits or ideas across a variety of available data sources (Peters et al., 2015, 2021). Therefore, a scoping review on the global condition of HE students’ EF research is relevant for this study.

This study implemented the Johanna Briggs Institute (JBI) approach, a comprehensive scoping review methodology framework (Peters et al., 2015, 2021) that builds on the initial guidelines of Arksey and O’Malley (2005) and earlier refinement of Levac et al. (2010), and applied to the following stages: *1. Defining and aligning the objectives and questions, 2. Developing and aligning the inclusion criteria with the objectives and questions, 3. Describing the planned approach to evidence searching, selection, data extraction, and presentation of the evidence, 4. Searching for the evidence, 5. Selecting the evidence, 6. Extracting the evidence, 7. Analysis of evidence, 8. Presentation of the results, and 9. Summarising the evidence in relation to the purpose of the review, making conclusions and noting any implications of the findings.*

A protocol was then developed priori to ensure alignment and the PRISMA extension for Scoping Reviews (PRISMA-ScR; Tricco et al., 2018) was used to capture the reporting phase of the review. Although the JBI guidelines did not prescribe registration as a compulsory requirement at the time, the document was nevertheless scrutinised and approved by an ethics committee of a reputable university.

### 2.1 Review questions and eligibility criteria

The participant, concept, context (PCC) method was employed to develop the eligibility criteria and to formulate the review question for this scoping review (Peters et al., 2020).

The following broad research question is posed: What is the scope and nature of scholarly research on undergraduate students’ EF skills in HE as published between 2014 and 2024? Sub-questions of specific interest are: How many relevant studies on EF skills in HE have been published? What types of research designs have been used in these studies? What key themes or focus areas have been addressed in the literature on undergraduate students’ EF skills?

Participants included undergraduate students of any geographical background, gender or race, who were enrolled for any diploma or degree qualification of any HE institution globally. The setting entailed academic contexts in HE. Due to the focus, the eligibility criteria excluded preschool, primary school, or high school learners as well as post-graduate students. The concept of primary interest was the EF skills of the participants. No eligibility criteria regarding a specific educational field were specified, but ultimately, health-related research was excluded to maintain clear focus, coherence, and consistency considering mapping studies in which EF serves as the primary concept of interest in academic contexts in HE. Table 1 summarises the inclusion and exclusion criteria that are described in the following sections.

**Table 1:** Inclusion and exclusion criteria.

Inclusion	Exclusion
Research published in 2014–2024	Other languages besides English
Articles published in peer-reviewed journals	The population of children in preschool, primary school, or adolescents in high school
Focus on students in higher education	Health-related research and/or behaviours
Any research design	Grey literature, reports, discussion pieces, reviews, opinions, and conference proceedings
Population of undergraduate students enrolled at a higher education institution	

#### 2.1.1 Search strategy and screening process

In line with the priori protocol, the inclusion criteria for this scoping review encompass research articles published in English between January 2014 and December 2024, a time when research regarding EF started to grow. This search period was selected to cover the last 10 years of research, enabling an evaluation of current trends, innovations, and gaps in the literature. This period was chosen to cover the last 10 years of research, which has seen a significant rise in interest in and studies of EF. By restricting the review to this timeframe, it is possible to evaluate current developments, trends, and gaps in existing research. Articles employing any research design, published only in peer-reviewed journals, were included, mitigating quality concerns. Although there are situations in which grey literature is advantageous, peer-reviewed publications in a scientific journal aid in ensuring that only the best quality of data is published (Johnson et al., 2025).

Boolean operators were identified to capture studies associated with the EF skills of undergraduate students in HE to discover peer-reviewed published studies. For each database and/or information source that was added, the search method was adjusted to account for different keyword and subject phrase descriptors: (“executive function*” or “executive function* skills” or “working memory” or “cognitive flexibility” or “inhibitory control”) AND (“higher education” or “tertiary education” or university or college) AND (student or “student teach*” or “preservice teach*” or “pre-service teach*” or practicum) AND (“academic achiev*” or “academic performance” or “academic success” or “education* success” or “student success” or “student achiev*”) NOT (“primary school” OR “elementary school” OR kindergarten OR “primary education” OR “elementary education” OR preschool OR “pre-primary school” OR “secondary school” or “middle school” or adolescent* or teenager* or child* OR childhood OR learner*) NOT (disab* OR impair* OR special OR “special needs” OR disorder OR disease OR patient* or health).

An initial incomplete search by the first author in Eric, EBSCOHost, PsychInfo, Web of Science, Scopus, and Taylor and Francis was undertaken between November 2023 to December 2024 to identify articles on the topic. The educational database, ERIC, was excluded from the final search since it was integrated as a secondary database in EBSCOHost. Therefore, the keywords contained in the titles and abstracts of relevant articles and the Boolean operators used to describe the articles were used to develop a full search strategy for EBSCOHost, Web of Science, and Scopus. Studies that appeared in bibliographies and within the text were added if they matched the inclusion and exclusion criteria.

Following the predetermined search, articles were primarily identified and uploaded into the citation software, Zotero, version 6.0.30 (2023), and duplicates were removed after being imported into Rayyan, which is software used to conduct systematic reviews. After a pilot investigation, the titles and abstracts were screened by two independent reviewers who compared them to the scoping review’s inclusion requirements. The reviewers reached an agreement on each article’s eligibility, and screening proceeded. Potentially relevant full-text sources were recovered and screened independently by the two reviewers. Conflicts that arose during both the abstract and title, and full-text screening procedures were resolved through discussion. If an agreement was not reached, a third reviewer would have been considered. Twenty-eight publications remained after the study selection process was completed. For several reasons, the eliminated research turned out to be irrelevant to this investigation. Many of the records that were discovered had no relevance to the topic that was discussed.

#### 2.1.2 Data extraction

Two independent reviewers extracted data from the identified studies using a Microsoft Excel extraction spreadsheet. A few studies were used for pre-testing to guarantee the form’s accuracy, uniformity, and validity. During this process, the data extraction tool underwent incremental modifications, including additional information (notes as new knowledge became apparent) relating to HE students’ EF skills. One reviewer extracted data from the eligible publications; the other reviewer verified the reliability and validity of the information collected. The two researchers individually coded the data applying the created coding system to guarantee accuracy. By comparing the coding findings, inter-rater reliability was established, and disagreements were handled until an agreement was obtained through discussion. Consensus among raters was demonstrated by a Cohen’s Kappa coefficient of 0.82. The information collected included the author’s details, year of publication, design, methodology, sample size, field of education, country of origin, limitations and topics of the studies. Table 2 summarises the information that was collected from the selected studies.

**Table 2:** Overview/description of study articles.

Article number	Author	Year of publication	Design	Methodology	Sample size	Field of education	Country of origin	Limitations of the studies	Topics
1	Durak	2023	Quantitative	Online Authentic Learning Self-Efficacy Scale, Cognitive Flexibility Scale, Creative Self-Efficacy Scale	*N* = 102	Psychology	Turkey	Personales variables like gender, age, and academic success have no effect. No significant effect of creative self-efficacy on online ALSE.	Self-efficacy, experiential thinking, cognitive flexibility
2	Looney et al.	2023	Quantitative	CCT programme Wide range assessment of memory and learning-2 (WRAML-2) Neuropsychological processing concerns checklist. Captain’s log cognitive training programme.	*N* = 50	Education	USA—California	Participants were predominantly Caucasian, limiting generalisability. Teachers were not blind to students who received training. The control group lacked an active control activity.	Academic success, cognitive functioning, teacher perceptions, teacher-ratings, working memory, EF, school based computerised cognitive training
3	Wang & Jou	2023	Qualitative & quantitative	Surveys and assessment forms Measurement of Self-directed Learning in Online Learners The Cognitive Flexibility Scale (CFS)	*N* = 50 *N* = 1,219	Engineering	Taiwan	No specific limitations were mentioned in the research paper.	Flipped classrooms; mobile learning; learning emotions; cognitive flexibility
4	Amzil	2022	Quantitative	Digit span test Regulation of cognition factor of the Metacognitive awareness inventory Cumulative GPA of the participants	*N* = 139	Education	Morocco	No specific limitations were mentioned in the provided contexts.	Working memory, cognitive regulation & monitoring, GPA, academic performance, meta-cognitive
5	Niazi & Adil	2021	Quantitative	Heart and Flowers Task Raven’s Standard Progressive Matrices™ Plus CGPA of students	*N* = 560	18 departments	Pakistan	No specific limitations were mentioned in the provided contexts.	Academic achievement, fluid intelligence, working memory
6	Toraman et al	2020	Quantitative	Cognitive Flexibility Scale (CFS) Quality of Faculty Life Scale (QFLS) Approaches to Learning Questionnaire (ALQ) Grade point average (GPA)	*N* = 1,573	16 faculties	Turkey	Path models are not confirmed by the goodness of fit indices. The goodness of fit indices did not meet the acceptable range criteria.	Cognitive flexibility, quality of faculty life, learning approaches, academic achievement
7	Gareau et al.	2019	Quantitative	Self-report measure A lexical decision task of AM and working memory tasks. Semester GPA	*N* = 258	Psychology	Canada	Gender moderates the relationship between fluid intelligence and academic achievement. Working memory positively predicts fluid intelligence and academic achievement. Fluid intelligence mediates between working memory and academic achievement.	Motivation, Achievement, implicit working memory, Self Determination Theory
8	Rosen et al.	2018	Quantitative	Webexec Media and Technology Usage and Attitudes Scale The daily smartphone usage subscale of the MTUAS Multitasking Preference Inventory GPA	*N* = 216	Education	USA—California	Study limitations include self-reporting, study length, and model structure.	Classroom digital metacognition, FOMO, Anxiety, EF problems, Academic performance, Multitasking, Attention, Smartphone use
9	La Lopa & Hollich	2014	Qualitative	Literature review	N/A	Culinary education	USA—Indiana	Working memory has limited storage capacity and is easily disrupted. Information in working memory can be lost due to decay. Individuals have varying working memory capacities.	Working memory, culinary arts, instructional design
10	Follmer	2021	Quantitative	Indirect measure: Executive skills questionnaire (ESQ) Direct measure: plus-minus task Category and verbal fluency tasks Letter memory task	*N* = 189	Educational psychology	USA—West Virginia	Calibration bias measures may not depend on the correlation between scores.	EF, metacognition, self-regulated learning
11	Corcoran & O’Flaherty	2017	Quantitative	Behaviour Rating Inventory of Executive Function Adult Version (BRIEF-A)	*N* = 376	Teacher Education	Ireland	Missing data and attrition impact sample estimation. Relies on self-report measures and lacks ability-based executive function measures. Study conducted in an Irish university, generalisation to other universities varies.	EF, pre-service teachers, teacher preparation
12	Cebi & Guyer	2022	Quantitative	Disorientation scale Academic achievement test Satisfaction scale Operation-word span task (OSPAN) Navigational metrics	*N* = 81	Scientific research methods” course. Computer Education and Instructional Technology degree	Turkey	Data was collected from a specific group of students in Turkey. Perceived disorientation measured subjectively, recommends objective methods for future studies. The study was limited to predicting disorientation, academic achievement, and satisfaction.	Working memory capacity, navigational behaviour, disorientation, academic achievement, satisfaction
13	Gareau et al.	2019	Quantitative	Irrational Procrastination Scale Coping Inventory for Academic Striving Computerised tasks (OSPAN and RSPAN) SGPA	*N* = 258	7 faculties: sciences, social sciences, health sciences, arts, medicine, management, and engineering.	Canada	Lack of experimental manipulation of psychological variables. Difficulty in creating experimental designs to manipulate mediating variables.	Procrastination, academic achievement, coping, self-regulation, working memory, EF
14	Ramos-Galarza et al.	2020	Quantitative	EFECO Scale self-report version Diagnostic criteria of the DSM-5 for attention-deficit/hyperactivity disorder adapted to a self-report scale Grades	*N* = 175	4 faculties: Psychology, environmental engineering, biotechnology, occupational health and safety.	Quito, Ecuador	The small sample size is not representative of the country.	Academic performance, Attention deficit, EF, Impulsivity
15	Ramos-Galarza et al.	2023	Quantitative	Executive functions scale for a university setting (UEF-1)	*N* = 1,373	Not specified (students studying at a private, state or municipal university).	Chile, Ecuador	Subjective bias in self-report evaluations generates participant behaviour appreciation bias.	EF, learning, behaviour, academic performance
16	Chen & Qu	2021	Quantitative	The Forward Digit Span Task paradigm Chinese Making Sense of Adversity Scale (CMSAS) Sub-scale—Positive and Negative Affect Schedule (PANAS) Three framing conditions (Opportunity, Risk, vs. Null)	*N* = 97	Technology	Singapore	Gender effects and underlying mechanisms need further examination.	Working memory, appraisal, challenge, threat, affect, framing,
17	Pinochet-Quiroz et al.	2022	Quantitative	Scale to assess the Executive Functions (EFECO) in its self-reported format Self-Regulated Learning Management Scale	*N* = 379	Students in pedagogy careers	Chile, Ecuador	The sample was by convenience, suggesting a larger, heterogeneous sample. Future research should include an analysis of hot executive functions.	Cold EF, EF, self-regulated learning, self-regulated learning management
18	Dias et al.	2022	Quantitative	Socioeconomic classification Scale Cloze test Reading comprehension task—The Ark at the End of the World (RCT) Adult self-report scale 1.1 (ASRS) Inventory of difficulties in executive functions, regulation and Delay Aversion for Adults Programme of Intervention in Executive Functions for Academic Learning (pFEx-Academics) Pre-test Intervention Post-test	*N* = 129 students *N* = 2 professors	Nursing, Physiotherapy, Nutrition, Psychology, & 2 Portuguese Language professors (Reading and Text Production teachers)	Saõ Paulo—Brazil	Absence of intelligence measures or known Learning Disorders diagnosis. The reliability of some measurements was only acceptable. Study conducted in a single Higher Education Institution.	Academic achievement, cognitive promotion, intervention, self-regulation, EF
19	Harel-Gadassi	2022	Quantitative	The Behaviour Rating Inventory of Executive Functioning–Adult Version The Self-Efficacy Questionnaire (SEQ) Academic Motivation Scale	*N* = 118	Professional studies, BA degree studies, MA degree studies, and PhD degree studies.	Israel	Transition to distance learning due to the pandemic impacted study results. Gender imbalance in participants due to sampling methods. The study focused on academic achievement, not student experience or preference.	EF self-efficacy, motivation
20	Pendry et al.	2021	Mixed methods	12 weeks participation: Baseline assessments (Week 1) 4 weeks of 1-h-long programme sessions (Weeks 2–5) Post-test assessments (Week 6) Hiatus of 6 weeks, follow-up assessments (Week 12)	*N* = 309	Students at risk—not related to a specific educational field	USA -Washington, Virginia	Study design strengths include random assignment and comparison methods. Used consistent facilitation staff, scripts, timing, and reviews to limit bias. Examined prolonged, regular exposures over 4 weeks.	Human–animal interaction, animal-assisted activity, EF, randomised controlled trial, stress prevention, at-risk students
21	Gee et al.	2015	Quantitative	Experiment iPad® application called Mimic Challenge Polar Pro Trainer ambulatory heart monitor	*N* = 31	Students from different majors; mostly psychology.	USA—New York	The study includes limitations and caution in interpreting findings.	Cognition, dog, heart rate variability, touch, working memory
22	Crouzevialle et al.	2015	Quantitative	Experiments Automated Operation Span task (OSPAN) Arithmetic problems	*N* = 96	Engineering, political and social sciences, arts and humanities, law, and business.	Switzerland	The study lacks monetary rewards and peer pressure elements.	Working memory
23	Chew et al.	2016	Quantitative	Experiments (reading comprehension, music)	*N* = 165	Arithmetic, language & music	Singapore	Violation of univariate normality assumption, but robust analysis continued.	Music, familiarity, language of lyrics, learning, task performance, EF
24	Knouse et al.	2014	Quantitative	Barkley Deficits in Executive Functioning Scale—Short Form (BDEFS) Grade point average (GPA)	*N* = 479	Psychology	USA	No specific limitations were mentioned in the provided contexts.	EF, GPA, goal setting
25	Santa-Cruz & Rosas	2017	Qualitative literature review	Literature review	Students in higher education (not specified)	Educational Psychology	Chile	The relationship between teachers’ EF and teaching effectiveness is unexplored.	Social cognition, EF, teaching effectiveness, theory of mind, empathy
26	Quarles et al.	2024	Quantitative	Big Five Inventory-44 and a psychological assessment battery, which included the Trail Making Test and the semantic Fluency Test	*N* = 200	Psychology	USA	Associations with extraversion, conscientiousness, agreeableness, and openness are lacking.	EF, conscientiousness; cognitive flexibility, neuroticism
27	Manuhuwa et al.	2024	Quantitative	Performance-based & self-report questionnaires	*N* = 748	Psychology	Netherlands	Results might not be generalisable. Evaluation of several variables can impact each participant’s cognitive abilities. Self-reported EF and performance-based EF assessments did not correlate.	EF, study success
28	Liao & Zhao	2024	Quantitative	Path analysis, factor analytic tests 64-card version of the Wisconsin Card Sorting Test (WCST-64) The Digit Span Task Location Span Task	*N* = 135	Communi-cation	China (Hong Kong)	Demographics: Most of participants were female. The online writing platform underrepresents the complexity of students’ writing habits.	EF, integrated writing, integration activities

#### 2.1.3 Data analysis and presentation

**Figure 1:**
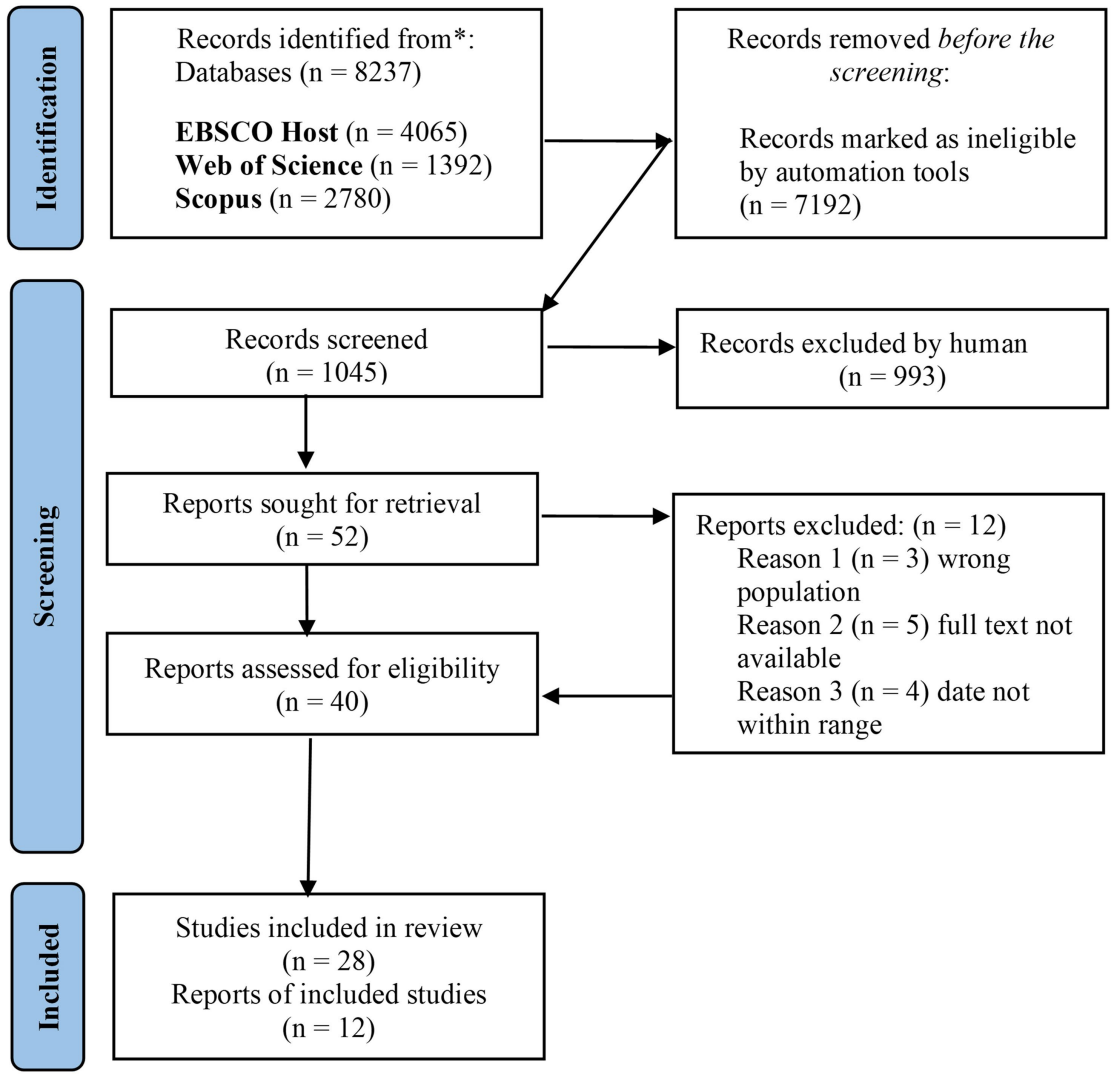
PRISMA flow diagram portraying the review process.

A descriptive qualitative content analysis method was employed to narratively synthesise textual information regarding the rationale behind the existing evidence of EF (Peters et al., 2020), especially in the HE setting. Initially, the generated data was prepared and structured into different categories, and sub-categories. The data was analysed inductively (from the data) and deductively (according to theory) and interpreted in which key patterns and themes were identified (Schreier, 2012). Tables, figures, and narrative descriptions of results applicable to the review’s objectives and questions are used to present the data (Schreier, 2012). This scoping review contains a comprehensive report on the search and research inclusion process results and is displayed in a Preferred Reporting Items for Systematic Reviews and Meta-analyses extension for scoping review (PRISMA-ScR) flow diagram (Page et al., 2021) in Figure 1. The flow chart shows how many records were identified, reviewed, eliminated, and added to the overall synthesis. This made it possible to record how the studies proceeded through each stage in a systematic manner. Critical appraisal was not conducted since the aim of this scoping review was to “map the available evidence rather than provide a synthesised and clinically meaningful answer to a question” (Peters et al., 2020, p. 2124).

## 3 Results

After a pilot investigation, we found it suitable to search three electronic databases for peer-reviewed published literature, resulting in 8237 articles. The abstract and title of 1,045 articles were screened after 7,192 irrelevant articles were excluded. After removing irrelevant articles, the two independent researchers reviewed, extracted and screened 52 full-text and peer-reviewed articles, yielding 40 articles that complied with the qualifying criteria. At the end of the screening process, 28 full-text articles remained. The excluded research was not relevant to this investigation for several reasons. Many of the records that emerged did not relate to the topic of the study. The study’s target population, articles published outside the specified range (2014–2024), and EFs based on health-related studies were common reasons for exclusion. In addition, some full-text articles were unavailable, despite the authors’ efforts to obtain them through the institution’s library and legal open access tools. According to the study criteria, grey literature, reports, discussion pieces, reviews, opinions, and conference proceedings were also ineligible. The PRISMA flow diagram portraying the review process is depicted in Figure 1.

This inaugural scoping review offers an overview of how executive function (EF) skills have been studied among undergraduate students in higher education, focusing on the volume, research designs and focus areas of existing studies.

### 3.1 Year characteristics: year of publication

**Figure 2:**
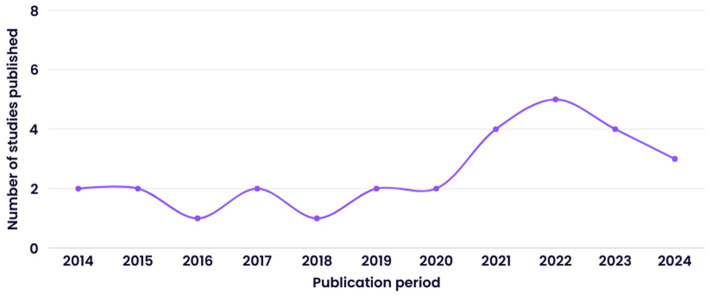
Distribution of studies by publication period.

This scoping review produced a total of 28 fully qualifying research studies in the publication period 2014 to 2024 and is illustrated by year of publication in Figure 2.

The line graph in Figure 2 indicates a sharp rise in scholarly attention in the last 3 years, as 16 of the studies were conducted between 2021 and 2024. This suggests that the topic is gaining traction and underscores the timeliness of this review. The emphasis on recent research further indicates that the identified constraints and practical consequences are based on contemporary findings. Including recent studies ensures the research reflects current trends and developments in education, providing the most recent information in the field.

### 3.2 Countries of origin of the included studies

**Figure 3:**
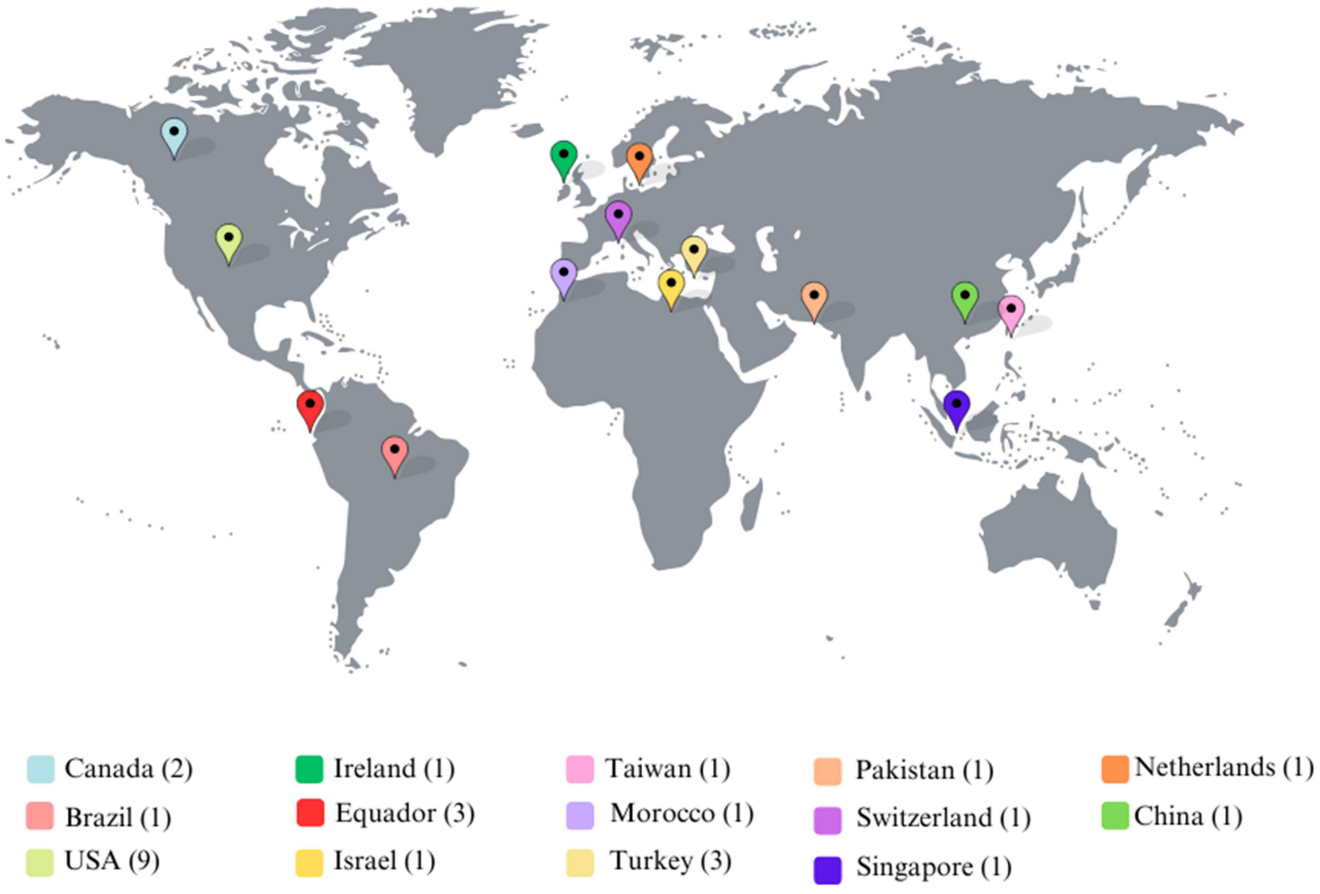
Country distribution of research conducted.

Figure 3 portrays the number of included studies conducted globally. The studies were conducted with undergraduate students in 14 countries—nine in the United States, three in Turkey and Ecuador, two in Canada and Singapore, and one each in Taiwan, Morocco, Pakistan, Ireland, Brazil, Israel, China, the Netherlands, and Switzerland. Research findings indicate a heavy emphasis on studies in the northern hemisphere (84%), while a lack of studies in the southern hemisphere (16%) is evident. Only one country, Morocco, is located on the African continent. This regional disparity reveals an obvious prevalence of studies conducted in western and northern locations, where HE systems tend to have greater resources and more sophisticated technology. In addition, the small number of global south studies, especially in Africa and regions of Asia and South America, raises the possibility that many HE students’ psychological, socioeconomic, and academic experiences are not adequately reflected in current research.

### 3.3 The methodology employed in the studies

Of the 28 included publications that met the inclusion criteria, only a small number, *n* = 2 (7%) employed a qualitative research design. The majority, *n* = 24 (86%), employed a quantitative design, and the remaining fraction, *n* = 2 (7%), used a mixed or multi-methods technique. EF can be evaluated using a range of methodologies, with the predominant ones being laboratory assessments (computerised cognitive training) and behavioural rating scales. These methodologies exhibit certain advantages and constraints in the evaluation of EF. Specifically, the employment of laboratory assessments raises an issue regarding the lack of uniformity between results and real-world scenarios, given the high artificiality of neuropsychological experimental tests (Ramos-Galarza et al., 2019). Students’ Grade Point Average (GPA) is commonly utilised as a method for evaluating academic performance, as evidenced by various articles (4, 5, 6, 7, 8, 13, 24). Solely one investigation (12) opted not to utilise GPA for this purpose. Diverse assessment tools, such as scales, assessments, surveys, and questionnaires, are employed to evaluate a range of components, including self-efficacy, CF, WM, EF, and academic achievement, among others. Experimental analyses were conducted in four instances (21, 22, 23, 28), whereas two studies (9, 25) comprise a literature review to consolidate discoveries on EF. The self-report questionnaires that were employed in the included studies are depicted in Table 3.

**Table 3:** Self-report questionnaires used to measure EF.

Self-report questionnaires	Description of the measurement scale
Metacognitive Awareness Inventory (MAI)	Assesses students’ cognitive control, particularly the metacognitive thinking (Amzil, 2022).
Behaviour Rating Inventory of Executive Function–Adult Version (BRIEF-A)	Measure EF in adults (Rabin et al., 2011).
Barkley Deficits in Executive Functioning Scale (BDEFS)	Measure EF deficits in university students (Knouse et al., 2014).
EFECO Scale (Executive Function Scale of Amsterdam)	Evaluates EFs used in the actual setting of a university student’s everyday life (Ramos-Galarza et al., 2019).
Executive Functions Scale for a University Setting (UEF-1):	Evaluate executive functions from a student’s behavioural point of view in an academic environment (Ramos-Galarza et al., 2023).
Webexec	Using the internet to measure EF challenges in HE students (Rosen et al., 2018).

Performance-based tasks are straightforward and objective assessments of core EFs (Follmer, 2021) implemented in the studies, as depicted in Table 4.

**Table 4:** Performance-based tasks used to measure EF.

Performance-based questionnaires	Description of the measurement scale
Digit Span Test	Assess WM capacity (Amzil, 2022).
CNSVS	Measure performance-based EFs (Manuhuwa et al., 2024).
Operation-Word Span Task	Assess WM capacity (Çebi and Güyer, 2022).
General Executive Function Task Performance	Measure performance using EF tasks (Quarles et al., 2025).

### 3.4 Sample size

The articles incorporated in this study have a wide range of sample sizes. Most studies (12 studies: 1, 4, 7, 8, 10, 13, 14, 18, 19, 23, 26, 28) fall within the 101 to 300 range. A smaller group of five investigations (2, 12, 16, 21, 22) fall within the category of over 100 participants. Furthermore, two investigations (5, 27) included individuals within the 501–800 range, while four research projects (11, 17, 20, 24) focus on subjects within the 301–500 interval. Three of the studies (3, 6, 15) had the potential to recruit more than 1,000 participants. According to this distribution, the majority of research employed medium-sized samples, which are often adequate for quantitative analysis but may limit wider generalizability. The reliability and validity of results are enhanced by larger samples, as demonstrated in studies 3, 6, and 15, indicating a growing shift to more reliable empirical methodologies.

### 3.5 Disciplinary focus

The investigations encompass a wide array of disciplines within education (2, 4, 8); psychology (1, 7, 14, 18, 21, 24, 26, 27); engineering (3, 13, 22); teacher education (11); culinary arts (9); educational psychology (10, 25); communication (28); and students pursuing careers in pedagogy (17). Additionally, several studies involve various disciplinary fields simultaneously (5, 6, 12, 13, 14, 18, 19, 22, 23). One study involves students classified as at risk (20), while another study does not specify the educational domain (15). EF development differs significantly across HE disciplines. Research in education usually employs applied approaches connecting EF to pedagogy, lesson planning, self-regulated learning, and interventions aiming to improve academic performance. In psychology, the field in which EF is mainly studied, literature focuses on the main EF components: inhibitory control, cognitive flexibility, and working memory, and how these relate to mental health, adjustment, and academic performance. Furthermore, EF is demonstrated in engineering by innovative problem-solving, conceptual thinking, and creative design, all linked to an immense amount of preparation, perseverance, and flexibility. Studies in communication: language and literacy, often investigates how EF facilitates the processing of information, interpersonal relations, and communicating ideas. Certain disciplines, such as culinary arts, are under-researched. However, the article included in this study reflects that EF development is crucial to follow instructions, perform calculations, analyse loads of information, and understand content, among other aspects. It is noted that the interdisciplinary focus highlights the complexity and interconnectedness of educational research, emphasising the potential for insights and collaboration across disciplines to address educational challenges.

**Figure 4:**
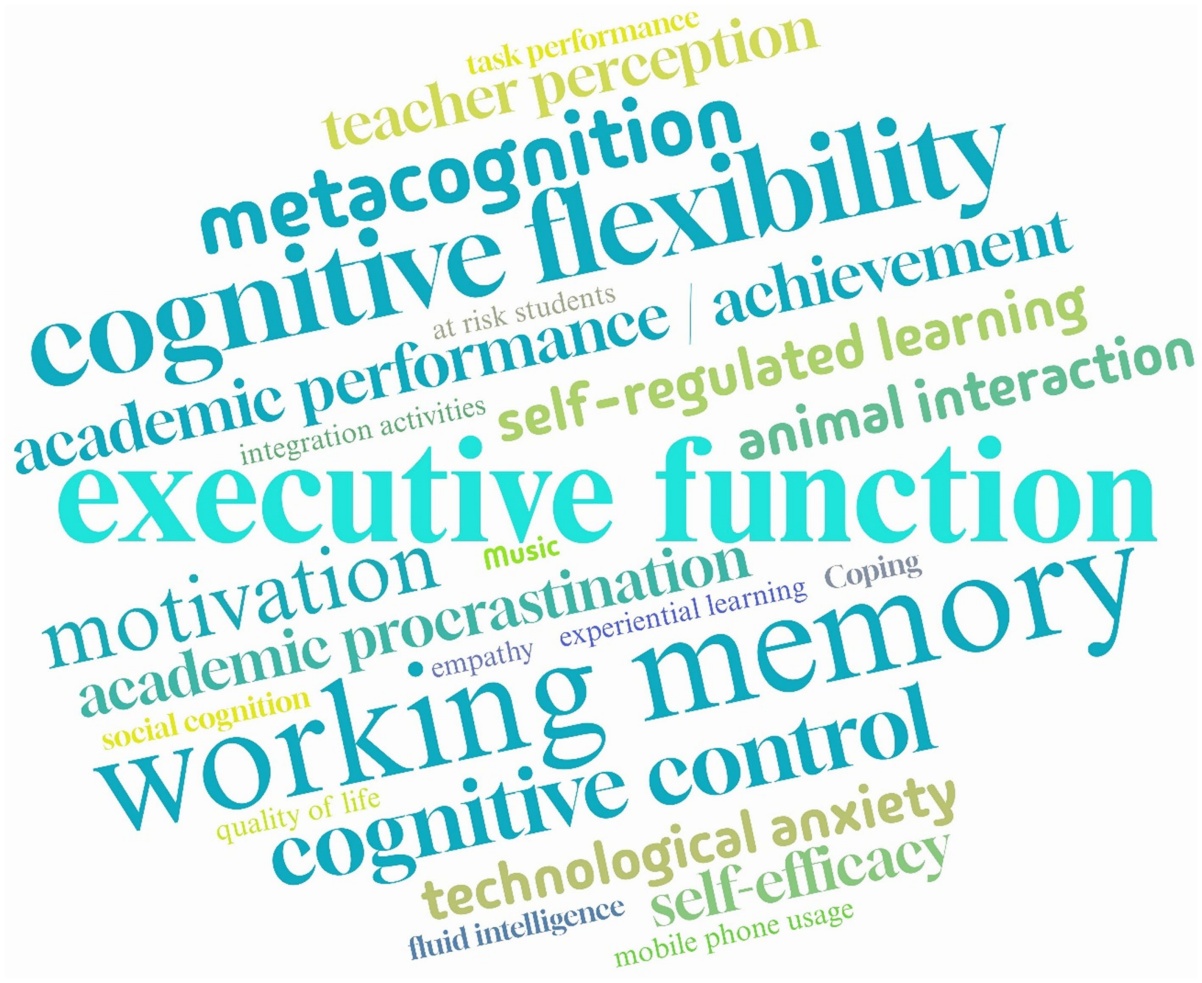
Visual representation of the concepts identified in the included articles.

Figure 4 depicts the topics outlined in the 28 included articles, linked to EF abilities among HE students.

EF, as an overarching construct, is referenced in 15 articles, while academic achievement/performance is linked to EF in 14 articles. Furthermore, nine studies focus on working memory and four studies on cognitive flexibility. Moreover, three articles, respectively, refer to metacognition, cognitive control, and motivation. Two articles examine EF in relation to combinations of self-regulated learning, technological anxiety, self-efficacy, animal interaction, and teacher perception. Finally, the following concepts appear once: authentic learning, at risk students, academic procrastination, coping, experiential learning, flipped classroom, fluid intelligence, mobile phone usage, music, quality of life, social cognition, task performance, integration activities, and empathy.

### 3.6 Addressing executive function challenges in HE

Numerous studies have advocated for developing efficient educational settings (1, 3), such as flipped classrooms (3) or enhancing instructional design, to promote student performance. Additional research has proposed implementing intervention programmes (10, 13, 14, 18) or assisting students facing cognitive or EF challenges (3, 15, 19, 20). According to the findings, identifying a student’s thinking style, flexibility, and self-efficacy serves as the foundation for creating an efficient learning atmosphere. A flipped classroom setting showed the greatest benefits for lower achieving students, whereas interventions might need to be modified for higher performing students. It is recommended that pedagogical approaches be adjusted to target cognitive flexibility (3), e.g., shift to a flipped classroom approach by creating customised lesson plans to cater for student needs and working memory (9), e.g., enhance students’ memory by breaking up content into smaller parts, provide numerous quizzes, and assigning many practice exercises on a specific topic to improve academic success. The assessment of EFs among students in HE has been suggested to promote academic achievement (15). Approaches focus on enhancing cognitive functions to produce better learning outcomes are acknowledged (2, 4, 17, 27). Studies have highlighted the positive impact of animal interaction on cognition (21), particularly among at-risk students (20). Motivation is recognised as an influential element in students’ academic success (7, 19, 24). Two research studies (25, 28) find that strengthening teachers’ EFs lead to enhanced teaching efficacy and improved academic pedagogical skills.

### 3.7 Executive functions and academic achievement

Academic achievement has consistently been associated with various intellectual and affective elements, including intelligence, motivation, self-efficacy, strategic behaviour, utilisation of cognitive and metacognitive strategies, resilience, and self-regulation, among other variables (Amzil, 2022). Pearsons correlation analysis was used to investigate these connections and regression analysis was applied to predict the potential of EF on GPA (Amzil, 2022). The research interest evident from the included studies in this review shows that it is primarily directed towards the academic performance of HE students, with a specific focus on the connection between EF and academic achievement. Of the scholarly articles, 14 explore this subject matter (2, 4, 5, 8, 12, 13, 14, 18, 19, 20, 22, 23, 24, 27). The direct association between EF and academic achievement implies that enhanced EFs are associated with improved academic success. On the other hand, the indirect relationship indicates that EF can impact academic success by affecting other psychological factors such as self-efficacy (1, 3, 19) and motivation (7, 19, 24). Article 19, specifically, delves into the connection between EFs and academic performance within the context of distance education. The article suggests that EFs not only exert a direct influence on academic success but also produce indirect consequences through variables such as self-efficacy, which pertains to one’s confidence in one’s abilities, and intrinsic motivation, which refers to the internal drive to acquire knowledge (Harel-Gadassi, 2022).

In addition, WM, a component of EF, is essential for academic achievement since it deals with the capacity to retain and manage information in the mind while completing activities like comprehension and problem-solving (Amzil, 2022). Various articles (4, 9, 14, 24) underscore the significance of EF, particularly WM, in influencing academic success. They contend that those who fail academically are more likely to have inadequate EF abilities, such as poor WM and trouble with cognitive regulation. Articles (2, 10, 14, 17, 20) concentrate on enhancing cognitive skills like WM, EFs, and CF to improve academic performance.

Furthermore, the connections between academic success and CF are explored in articles (1, 3, 6). They emphasise the relevance of CF across various learning situations, such as flipped classrooms and online social networks, and highlight it as a strong predictor of academic success. CF allows students to adapt to changing circumstances, correct mistakes, and seize opportunities (Diamond, 2013), enabling them to choose effective learning techniques and problem-solving approaches in education (Durak, 2023). CF can assist students in efficiently navigating complex topics and varied opinions in online social networks, and better connect with learning content and use it in interactive activities in flipped classrooms (Wang and Jou, 2023).

### 3.8 Importance of teacher perceptions and training in student-teacher interactions and academic achievement

Two studies report on teacher perceptions (2, 25) to impact HE students’ academic achievement. Teachers play a crucial role in shaping the educational setting and enhancing students’ achievements through various means, such as imparting subject knowledge, guiding the learning process, and offering academic assistance (Looney et al., 2023). To increase teaching efficacy and deepen the relationships between teachers and students, article 2 emphasises the need for teacher preparation programmes, improving teachers’ social cognition and EFs, an indicator of effective pedagogy. In addition, effective teachers can comprehend the ideas and intentions of students and perceive and react to students’ emotions, which helps them become more cognizant of their needs and viewpoints.

In article 25, teachers reveal factors impacting HE students learning, such as the relationship between the teacher and students, understanding each other, sharing views, and collaboration between them to achieve a common goal. The study reveals that when teachers possess strong EF skills, they can incorporate student feedback, evaluate their own teaching methods, and modify their approaches to accommodate a variety of student needs (Correia and Navarrete, 2017). This study indicates that students should be aware of the interactions with their teacher to ensure effective learning takes place.

### 3.9 Technology

Two articles (8, 12) discuss the need for flexible educational systems to meet diverse student needs and promote metacognition regarding technology use, with varying implications based on their research findings. Article 8 suggests strategies to enhance metacognitive awareness and reduce technology-related anxiety; Article 8 suggests strategies to enhance metacognitive awareness and reduce technology-related anxiety; developing students’ EF, self-regulated learning, and technological cognition, which may assist in minimising the poor academic impacts of technology overuse in HE (Rosen et al., 2018). The authors reported that regular cellphone use, and poorer digital cognitive ability were observed in students with lower EF, which resulted in lower academic results and reduced study concentration. Article 12 advocates that students’ WM capacity influences their ability to learn within technological environments, pointing to the necessity of flexible multimedia systems that offer specific guidance to lessen cognitive strain and improve learning outcomes in HE settings (Çebi and Güyer, 2022).

### 3.10 Motivation

Three articles (7, 19, 24) delve into the correlation between motivation and academic success, the significance of cognitive elements, and the pivotal role of self-motivation in education. While each article addresses EFs and motivation, they give preference to distinct variables within these frameworks. Article 7 studies the interplay between explicit and implicit motivation alongside WM capacity. Empirical evidence in the study highlights that WM capacity controls the interaction between implicit- and explicit motivation, which is essential for academic performance. With higher WM capacity, students are more equipped to combine their academic goals (explicit) and motivation desires (implicit) resulting in organised, focused, and goal-directed behaviours. In contrast, lower WM capacity leads to a decline in self-control and academic achievement. Article 19 emphasises the intermediary role of EFs through self-efficacy and intrinsic motivation, especially in online education. According to the study, EF has a direct correlation with academic success in distant learning and an indirect correlation with self-efficacy and intrinsic motivation. Consequently, greater self-efficacy increases students’ intrinsic motivation to study. This is especially relevant for students who have greater EFs: planning, focused, organised, goal-directed, and self-regulated. Lower EF, on the other hand, exhibits less intrinsic motivation and self-efficacy, which leaves students more susceptible to failure (Harel-Gadassi, 2022). Article 24 underscores self-reported deficiencies in executive functioning and issues with self-motivation as indicators of academic achievement. In essence, these articles concur that motivation influences academic performance.

### 3.11 Gender differences, fluid intelligence and academic achievement

Fluid intelligence is a core cognitive ability involving the use of abstract reasoning to address non-traditional problems, essential for academic success (Niazi and Adil, 2021). Previous research establishes that fluid intelligence predicts WM (Smolen and Chuderski, 2015). Two articles (5, 14) examine the gender disparities in WM, fluid intelligence, and academic achievement. These articles suggest that gender moderates the relationship between cognitive aspects and academic success, resulting in different outcomes for males and females. It is reported that gender differences in cognition are context dependent. Research conducted in article 5 reports that girls had higher levels of WM and academic achievement and a stronger association between fluid intelligence and academic success than boys (Niazi and Adil, 2021). The results of the studies are consistent with research showing that female students frequently demonstrate higher levels of autonomy and mental awareness, which could enhance the advantages of cognitive abilities like WM and reasoning skills.

### 3.12 Limitations of included studies

The studies listed in Table 2 reflect a wide range of limitations identified by the authors. These limitations relate to sample demographics, methodological constraints, and potential biases. Some studies mention limitations related to the representativeness of the sample (2, 14, 28) where the sample did not accurately represent the entire population studied. Other studies highlight methodological limitations (1, 2, 14, 10, 11, 19, 27), such as the absence of experimental manipulation or the reliance on self-report measures. These limitations demonstrate that most of the existing research is derived from self-reported information and convenience samples, which could impact the study’s reliability and generalizability. Limitations were not pointed out in four studies (3, 4, 5, 24). The insufficient awareness of limitations indicates that this field of research lacks critical reflection in several studies.

### 3.13 Factors affecting EF

A notable study conducted by Dias et al. (2022) identifies a gap in research where the majority of research on EF interventions addresses young children and adolescents (Diamond and Ling, 2016) and a lack of studies in adults. Dias et al. (2022) aim to assess the effectiveness of an EF intervention programme on the academic learning of university students to alleviate difficulties related to EF. Their findings suggest that integrating interventions aimed at EF in the HE setting can yield diverse advantages for students, such as enhanced conduct and written language processing, increased attention, hyperactive behaviours, and enhanced reading comprehension skills crucial for academic achievement (Dias et al., 2022). Furthermore, two studies (10, 13) propose implementing interventions related to EF skills among students to promote academic success. Although there are some areas where the articles overlap, each one offers unique recommendations for interventions or approaches that are suited to distinct facets of education. Examples of these include cognitive training (article 2), flipped classrooms (article 3), coping mechanisms (article 13), and programmes that involve animal interaction (articles 20, 21). When creating interventions to assist student performance, various articles (5, 11, 13, 19) emphasise the significance of taking individual differences, such as gender, EF levels, and cognitive abilities, into account.

## 4 Discussion

This study aimed to explore what has been revealed by research on EF in terms of knowledge gaps, study designs, types of measurements, and contextual factors in the HE context globally.

### 4.1 Academic success

The data derived from the studies consistently demonstrate that EFs are crucial for academic success in HE, significantly impacting students’ achievement. In addition, the studies report on the correlation between EF elements and academic success and although there are strong links across academic success and EF skills, the study’s non-experimental design restricts the ability to draw conclusions about causality. The articles discuss strategies for improving academic performance, including individual focus, targeting different areas of EF, and multiple interventions, with various concepts contributing to positive or negative effects. Academic success results in students adapting to the HE demands of flexibility, efficient time management, problem-solving, multitasking, attention and behaviour control, memory use, success management, and self-regulated learning; all linked to the three EF categories: IC, CF, and WM. EFs are the fundamental building blocks of self-regulated learning (Pinochet-Quiroz et al., 2022); a requirement to function well in HE.

A lack of development in the latter causes EF deficits, probably resulting in academic failure. HE institutions need to understand students’ challenges and EF abilities to ensure they employ suitable intervention strategies to enhance their chances of academic success. Low EFs are linked to poor academic achievement (Ramos-Galarza et al., 2019), which puts EF assessment in context as a useful tool for identifying students who may have learning difficulties and for institutions to understand the reasons behind them (Knouse et al., 2014). Since the prefrontal brain regions continue to develop throughout early adulthood, the research recognises that EFs remain flexible and may be enhanced and maintained (Dias et al., 2022) in HE. The articles reveal that WM is a key component in learning since it entails the cognitive space required for planning and recalling information. However, WM is characterised as constrained and susceptible to overload, particularly from too much information or interruptions (La Lopa and Hollich, 2014). In contrast, the authors state that the relationship between working memory and academic success can be mediated by fluid intelligence.

These studies address the impact of EF on HE students’ behaviour, academic performance, and autonomous learning, especially in the areas of neuroscience and educational efficiency, making substantial, unique additions to existing research (Dias et al., 2022; Pinochet-Quiroz et al., 2022; Ramos-Galarza et al., 2019).

### 4.2 Methodology

The evaluated research studies demonstrate diverse methods of measuring, mainly self-report scales, assessing challenges in daily life and performance-based assessments measuring cognitive skills through controlled activities (Follmer, 2021). The restricted connection between these two assessment tools is noteworthy, indicating that they represent separate components of EF (Manuhuwa et al., 2024). The authors assert that integrating self-reported and performance-based EF assessments could provide valuable insights into academic achievement. Research continuously shows that self-report assessments of EF and performance-based ratings do not significantly correlate, resulting in poor accuracy (Manuhuwa et al., 2024). The validity and main benefit and limitation of performance-based and self-report measures are highlighted in Table 5.

**Table 5:** EF measurement appraisal.

Type of assessment	Benefits	Limitations	Validity
Self-report measure	Since data is collected in day-to-day living, assessments are valid and realistic.	Contain personal element that may cause bias in the respondent’s assessment.	Display little correlation and no significant connections with performance-based EF tests.
Performance-based measure	Objective assessments of the main EF components (WM, CF, IC).	Activities are different from actions in real life.	Display little correlation and no significant connections with self-report EF tests.

Ultimately, the studies show that relationships between constructs can have positive or negative effects. Some articles investigate the EF-related constructs as dependent and independent variables, and others were tested as moderators.

### 4.3 Gaps in the studies

The publications included in this scoping review revealed certain gaps. Biographical disparities, such as gender differences, are not continually considered when measuring EF. The dominance of the northern hemisphere studies, particularly from the United States and parts of Europe, likely reflects both systematic and structural factors. First, major academic databases such as Web of Science, Scopus, and EbscoHOST, which we use as our sources, tend to index journals from institutions with high publication output, which are disproportionately located in the global north. Second, research findings and infrastructure in these regions often make large-scale, publishable EF studies more feasible. However, the lack of southern hemisphere representation is a significant gap, and it raises concerns about the global generalisability of findings and even the cultural validity of EF measures. One of the review’s key implications is an urgent need to encourage and amplify context-specific EF research in underrepresented regions, including Africa, Latin America and parts of Asia.

### 4.4 Contextual factors

Many of the interventions currently in use, such as computerised cognitive training or flipped classrooms, are often developed in Western academic settings. While this may show efficacy in this context, the impact elsewhere depends heavily on how well they align with the local educational culture, language practices, technology access, and student needs. Ensuring contextual appropriateness means engaging with local educators, adapting intervention materials linguistically and culturally, and evaluating outcomes within the specific socio-cultural environment. We also need collaborative research methods, particularly participatory approaches that involve stakeholders from diverse regions in both the design and implementation of the phases, because it is important that, as researchers, we must acknowledge that EF is not a ‘one size fits all’ construct. Its expression and development can be deeply shaped by cultural expectations, classroom dynamics, and even the role of the teacher-student relationship.

Implement sustained inclusive interventions, promote interdisciplinary collaboration and broaden research design across various types. To strengthen EF means equipping students with the required skills they need to excel by improving their ability to organise, prioritise, control mental load, and adjust to challenging situations in everyday life and in their studies. Finally, this review emphasises that EF elements like WM, IC, and CF are associated with academic achievement.

## 5 Limitations

This scoping review did not consider theses and dissertations, working papers, reports, and unpublished works, which could have caused bias. Future studies could reduce bias by incorporating unpublished work. Only studies released from the beginning of 2014 until December 2024 were included. Research undertaken at preschool, primary school and high school levels was excluded from this analysis. A few omissions might have occurred from the above criteria. Therefore, future research could conduct scoping reviews addressing the younger age groups and extending the search after 2024. Furthermore, this study focused only on the EF skills of undergraduate students in higher education. To address this limitation, future research on scoping reviews might examine interventions that enhance the EF of undergraduate students in HE. Additionally, since a scoping review does not necessitate it, this study did not formally assess the reliability of the evidence presented (JBI, 2015). This could compromise the findings’ credibility.

The methodological decision to perform a scoping review rather than a systematic evaluation presents a few implications. Rather than assessing the efficacy of interventions or synthesising outcome evidence, the review’s goal was to map and conceptualise existing EF research within HE in accordance with JBI guidelines. As a result, the review excluded essential components of systematic reviews: formal critical appraisal of study quality, bias risk, and analytical synthesis. A systematic review is therefore suggested for future studies on this topic.

A variety of specialised search phrases were used in the bibliographic databases for this scoping review. The topic-keyword combinations were selected to capture the EFs in HE that serve as the foundation for this review. Nevertheless, it is not impossible that other research that may have been relevant and that evaluated related phenomena but with different terminology may have been overlooked. Therefore, bibliographies of relevant publications have been screened to mitigate this limitation. It is recommended that future studies use citation monitoring tools to improve comprehensiveness.

## 6 Implications

### 6.1 Theoretical implications

The theoretical implications of EF are insightful incorporating a wide range of fields, including psychology, learning, and cognition. This scoping review was influenced by theoretical views on EF as a collection of mental functions to regulate thoughts, emotions, and behaviour (Diamond, 2013; Miyake et al., 2000). The literature highlights some important theoretical implications, especially the connection between EF, self-regulated learning, and academic performance. The core EF components such as IC help students maintain focus in online studying, while WM and CF allow them to adjust to challenging assessments.

Research emphasises the importance of EF for academic success, challenge preexisting theories, highlight causal relations, and provide new perspectives for improvement of EF. It is noteworthy that EF is even more important in today’s technological and independent-learning environments, where inadequacies surface as procrastination, poor self-control, and decreased success. The results of this review expand our knowledge of EF by presenting it as the complex control system responsible for planning, organising, goal setting, and self-regulation (Manuhuwa et al., 2024) among many others. Prospective educational legislation and guidelines should acknowledge EF as a fundamental competency that influences student achievement, engagement, and ability for continuous growth, rather than just as a psychological term.

The little overlap between the two kinds of measurements is a significant conceptual and practical challenge. Performance-based measurements in controlled laboratory environments assess hidden cognitive capacities while self-reports record achievement of objectives or behavioural expression in everyday, informal situations (Manuhuwa et al., 2024). For this reason, focused efforts to improve EF may be an important part of institutions to retain students. To create a holistic view, subsequent theoretical studies should develop a framework that incorporates performance-based and self-report measurements to assess undergraduate students’ EF skills. Consequently, these insights will enhance learning and teaching approaches, student support, and guidelines within HE.

### 6.2 Practical implications

Research highlights the practical implications and importance of understanding the relationship between self-efficacy, intrinsic motivation, and academic achievement in diverse HE settings for effective interventions and support structures. It is recommended that HE institutions employ a holistic approach to promote EFs and not focus on enhancing a single skill. The process of developing several EF skills will probably require a longer timeframe to produce advantages compared to focusing on enhancing a single skill. The former mentioned method proves to be more effective. For HE institutions, the vast amount of research on EF, namely WM and self-regulation, offers broad, practical implications. The implications focus on identifying students who are at risk, revising teaching strategies to lessen mental strain, and creating focused intervention programmes such as the pFex-Academics to enhance students’ EFs (Dias et al., 2022).

Instead of presuming students have sufficient self-regulation and EF abilities when they first enrol, institutions should take a proactive, evidence-based strategy regarding EF by implementing self-report evaluations to identify at-risk students (Manuhuwa et al., 2024). Teaching students to establish achievable goals and creating short tasks based on frequent due dates and lecturer input are proven methods to assist students who encounter EF difficulties like initiation, time management, and organisation (Rabin et al., 2011). Moreover, enhancing intrinsic motivation and self-efficacy are valuable motivational components of support to students with low EF abilities, especially in distance learning (Harel-Gadassi, 2022).

Another practical implication refers to the use of technology. Lecturers should consider using AI chatbots to serve as digital assistants and cognitive support tools to decrease cognitive strain, and improving critical thinking and CF (Pergantis et al., 2025). However, institutions should provide guidance and assistance to promote the ethical and responsible use of artificial intelligence (AI) to reduce plagiarism, falsification, and algorithmic information.

In education, practical implications focus on cognitive development, instructional design, creating efficient learning environments, and individual differences to enhance academic achievement and student success. These practical implications guide lecturers and policymakers in improving teaching and learning in demanding HE contexts, ensuring evidence-based institutions and effective policies for student success. Focusing on inclusive and varied methods of instruction will ensure that students’ cognitive and socioeconomic requirements are met. Finally, by drawing on interdisciplinary knowledge and expertise from different fields: psychology, education, and neurology, researchers can gain a more comprehensive understanding of educational issues and develop innovative solutions to improve EF, a requirement to function well in academia and life in general.

## 7 Conclusion

In this scoping review, 28 research articles about the nature and volume of EF in HE students worldwide were reviewed. A scoping review on the global condition of HE students’ EF research was relevant since EFs significantly impact their behaviour and performance. The authors discovered that during the previous 5 years, the number of studies in EF research in HE has increased significantly. This study demonstrates that the literature’s nature mainly includes topics on academic achievement, performance, and success. Furthermore, this review reveals how the literature’s scope encompasses student competency areas that cover all mental abilities, such as meta-cognition, cognitive control, working memory, and cognitive flexibility. Hence, other topics playing a role in HE students’ EF skills development include self-regulated learning, self-efficacy, motivation, technological anxiety, teacher perception, and animal interaction. This scoping review offers insight into existing literature by mapping and synthesising EF data on study designs, strategies, and essential concepts among undergraduate students in HE. The research published in the peer-reviewed publications of this review involved gathering, evaluating, and sharing data related to EF and reflects a quantitative study design. The results of this review could be of use in EF education, training, further research, and the development of policies and procedures. Based on policy, academic and instructional guidelines should acknowledge EF growth as a crucial element. In addition to being a psychological objective, enhancing EF in HE is a pedagogical requirement that promotes self-regulated learning, resilience, and academic performance. Therefore, HE institutions should integrate capabilities related to EF into lecturer development and student courses. To conclude, the results highlight how important EFs are for thriving in self-regulated learning and managing the mental requirements of HE. Despite the difficulties in reliably assessing these skills using various approaches, the research shows how important they are for learning and offers various options for guidance and encouragement to help students develop these vital strengths.

## Data Availability

The data supporting this review’s findings are available on request from the author LP.
